# Save A Leg – Exploring Disparities in Community Characteristics Between Regions of High and Low Leg Amputation Rates

**DOI:** 10.23889/ijpds.v9i5.2701

**Published:** 2024-09-18

**Authors:** Chuanfen Ni, Kyle Holtzman, Laura Minardi

**Affiliations:** 1 Northwestern University; 2 Google; 3 Oak Street Health

## Objective

To explore the disparities in community characteristics between the regions with the highest and lowest leg amputation rates.

## Approach

This study used an exploratory method to describe the disparities between community characteristics of these two regions. The study team used several data sources: 1) We used the Dartmouth Atlas Healthcare Map to identify the highest and lowest leg amputation areas. This data was based on leg amputations per 1,000 Medicare enrollees by Hospital referral regions (HRRs) in 2015. 2) We used population estimates and income data from The Census Bureau. 3) We gathered climate, crime index, walk score, bike lanes, and park data from numerous websites.

## Results

Florence, SC has the highest leg amputation rate and Bridgeport, CT has the lowest across the nation. Florence also shows higher cardiovascular disease and diabetes discharge rates. Florence’s population has a slightly higher percentage of persons 65 years and over, a higher percentage of bachelor’s degrees, a higher per capita income, and a lower poverty rate. Florence also has fewer parks, is less walkable or bikeable, and has worse crime. It is worth noting that Bridgeport is close to Yale New Haven Hospital, which has a Limb Preservation Program that could contribute to the region’s extremely low leg amputation rate.

## Conclusions

There are disparities in community characteristics between the regions with the highest and lowest leg amputation rates.

## Implications

Future work needs to investigate the association between community characteristics and leg amputation rates and assess the impact of the Limb Preservation Program.

